# Supplementary material for: Functional characterization of cooperating MGA mutations in RUNX1::RUNX1T1 acute myeloid leukemia
Source: Leukemia. 2024 Mar 7;38(5):991–1002. doi: 10.1038/s41375-024-02193-y (PMC11073986; doi:10.1038/s41375-024-02193-y)
Supplement: Supplementary file 1 — Supplemental - Functional Characterization of Cooperating MGA Mutations in RUNX1-RUNX1T1 Acute Myeloid Leukemia. [file 41375_2024_2193_MOESM1_ESM.docx]

**Supplemental data**

**Methods.**

***Lentiviral Production.***

Lentivirus and retrovirus were generated as previously reported^1,2^. Briefly, HEK293T cells were plated at 5x10^6 cells/ml in DMEM with 10% FBS in a 10cm dish for ~40% confluency. After adhering to the plate, cells were transfected using the transfection cocktail containing FuGENE HD (Promega, WI), packaging plasmid mixture (pHDM-G, PCAGG-HIVgpco, pCAG4-RTR2 for lentivirus) or (Eco PAC for retrovirus), and the plasmid of interest (CL20c-MIG-DEST[nFlag-MGA], [nFlag-MGA-ex14-23], or [nFlag-MGA-C623*]) and pL-CRISPR.EFS.GFP [Mga guide] or [nontargeting guide]) for lentivirus and MSCV-IRES-GFP[RUNX1::RUNX1T1 9A] for retrovirus and incubated for 48hrs at 37C. Virus was harvest by filtering the virus-containing supernatant through a 0.2um filter. Lentiviruses were additionally concentrated to 500uL at 2000xg using an Amicon Ultra-15 Centrifugal Filter Unit (Millipore-Sigma, MA). pL-CRISPR.EFS.GFP was a gift from Benjamin Ebert (Addgene plasmid #57818)^3^.

***Viral Transductions***

For lentiviral transductions of MOLM-13 cells, 1 million cells were plated in a 6-well dish with 2.5mL fresh media (IMDM with 10% FBS), 1ug/mL polybrene, and 500uL of the concentrated virus described above. Cells were spinfected at 2000g for 1.5 hours at 30C. Cells were then incubated overnight at 37C. Spinfection was repeated and incubated for 4-6hrs at 37C then washed and replaced with fresh media. Transduced cells were then sorted for GFP+ cells 48hrs post spinfection using the FACSAria sorter (BD Biosciences, CA).

For lentiviral transductions of CD34+ cells, ~250K cells were plated in a 24-well dish with 1ml of fresh media (StemSpan SFEM II supplemented with IL-6, Flt-3 Ligand, SCF, TPO, StemRegenin1, and UM729). Spinfection was done as described above. For retroviral transductions of harvested murine HSPCs, 1 million cells were plated in a 12well dish with 1mL fresh media (RMPI with 15% FBS supplemented with murine cytokines including IL-3, IL-6, SCF, Thrombopoietin, and Flt-3 (PeproTech, NJ) as previously reported, 1ug/mL polybrene, and 1mL of the harvested retrovirus described above^4,5^. Cells were spinfected at 2000g for 1.5 hours at 30C then incubated overnight at 37C. Spinfection was repeated and incubated for 4-6hrs at 37C then washed and replaced with fresh media. Transduced cells were then sorted for GFP+ cells 48hrs post spinfection where indicated.

***In vivo transplantations***

For non-competitive serial transplants, 500,000 lineage-negative cells from harvested from donor mice, as described above, with 100,000 support whole marrow cells from CD45.1/.2 were injected via tail-vein (I.V.) into lethally irradiated age and sex-matched CD45.1 recipient mice. For secondary transplants, WBM was harvested from primary recipient mice at 16-weeks, and 1 million cells were injected (I.V.) into lethally irradiated age and sex-matched CD45.1 secondary recipient mice.

For *RUNX1::RUNX1T1* transplants, 500,000 lineage-negative cells were harvested from donor mice 48 hours post transductions, as described above, and 100,000 support whole marrow cells from CD45.1/.2 were injected via tail-vein (I.V.) into lethally irradiated age and sex-matched CD45.1 recipient mice.

***CFU Assays***

Lineage-negative cells isolated from primary mice or from sorted *RUNX1::RUNX1T1* expressing cells were cultured in MethoCult™GF M3434 medium (StemCell-Technologies, Canada) at 3,000 cells/mL and incubated at 37°C with 5% CO2 for 7 days then colonies were counted. For serial replating, colonies were harvested by washing in PBS and were re-cultured in M3434 medium (10,000 cells/mL for primary cells and 3,000 cells/mL for *RUNX1::RUNX1T1* 9A cells) for additional 7 days for up to five rounds.

***CRISPR/Cas9 Knockouts***

*MGA* knockout MOLM-13 cells were generated using CRISPR-Cas9 technology. MOLM-13 cells were transfected via nucleofection (Lonza, 4D-Nucleofector™ X-unit) with ribonuclear proteins (RNPs) consisting of 100 pmol of sgRNA (ACTGGAATCAACAACAATCGNGG) and 33 pmol of spCas9 protein. Transfected cells were single-cell sorted into 96-well plates and expanded. Expanded clones were sequenced for in-frame indels of *MGA* via targeted deep sequencing as previously described^1^.

***Histology***

Histology was done as previously described^5^. Briefly, following euthanasia, animals were placed in 10% neutral buffered formalin. The bones were collected and subsequently decalcified in 10% formic acid. The tissues were then embedded in paraffin and 4-um sections were stained with hematoxylin and eosin (HE).

***Flow cytometry***

Analytical flow cytometry was done using LSR FORTESSAII (BD Biosciences, CA). Immunophenotyping on WBM, spleen, and blood was performed using cell surface fluorescently labeled antibodies and viability was assessed using DAPI (**Supplemental Table 1**). For intracellular staining with Ki67, indicated cells post-immunophenotyping were fixed with 4% paraformaldehyde, permeabilized with 0.5% Triton X-100, and stained with Ki67 and NuclearMask (Invitrogen, CA) (**Supplemental Table 1**). EdU-incorporation assay was done using the Click-iT Plus EdU Kit (Invitrogen, CA) following the manufacturer’s protocol as previously described^1^.

***Fluorescence Microscopy.***

HEK293T cells were transfected as described above with GFP-tagged MGA and MGA truncations and platted on poly-L-lysine coated coverslips for 24hrs. Cells were then fixed with 4% paraformaldehyde, permeabilized in 0.3% Triton X-100, blocked with 5% rat serum, and stained with the indicated antibodies and DAPI (**Supplemental Table 1**). Coverslips were mounted onto slides using ProLong Diamond Antifade (Invitrogen, CA). Images were acquired on a Nikon C2 laser scanning confocal microscope at 60X using an oil-objective lens (Nikon, Japan).

***IP-Westerns***

HEK293T cells were transiently transfected as described above with flag-tagged MGA constructed. Cells were harvested in PBS and then lysed with 150mM NaCl, Tris-HCl pH8, 1% NP40, and 0.5% sodium Deoxycholate with a protease/phosphatase inhibitor. Lysates were then incubated on Anti-DYKDDDDK Magnetic Agarose (ThermoFisher, MA) for 4hrs at 4C^O^ with rotation. Agarose was washed 3X using lysis buffer and proteins were eluted with 150mM NaCl, Tris-HCl pH8, 2% SDS, 10% glycerol, and 0.5% Triton X-100 with boiling.

**Supplemental Table 1:** Antibody reagents

|  | **REAGENT or RESOURCE** | **SOURCE** | **IDENTIFIER** |
| --- | --- | --- | --- |
|  | **Antibodies** | | |
| **Mature Panel** | BV605 anti-mouse/human CD45R/B220 | Biolegend | 103244 |
|  | PE/Cy7 anti-mouse CD3ε | Biolegend | 100320 |
|  | PE anti-mouse/human CD11b | Biolegend | 101208 |
|  | PerCP-Cy5.5 anti-mouse Ly-6G/Ly-6C (Gr-1) | Biolegend | 108428 |
|  | PerCP-cy5.5 anti-mouse CD71 | Biolegend | 113816 |
|  | AF488 anti-mouse CD45.1 | Biolegend | 110718 |
|  | BV650 anti-mouse CD45.2 | Biolegend | 109836 |
| **HSPC Panel** | PerCP-Cy5.5 Ly-6A/E (Sca-1) Monoclonal Antibody (D7) | Thermo Fisher | 45-5981-82 |
|  | PE/Cy5 anti-mouse CD127 (IL-7Rα) | Biolegend | 135016 |
|  | PE/Cy7anti-mouse CD34 | Biolegend | 128618 |
|  | AF700 anti-mouse CD48 | Biolegend | 103426 |
|  | APC-ef780 c-Kit (2B8) | eBioscience | 47-1171-82 |
|  | BV605 anti-mouse CD150 | Biolegend | 115927 |
|  | BV711 CD16/CD32 | eBioscience | 56-0161-82 |
|  | AF488 anti-mouse CD45.1 | Biolegend | 110718 |
|  | BV650 anti-mouse CD45.2 | Biolegend | 109836 |
| **Lineage cocktail** | PE anti-mouse CD3ε | Biolegend | 100308 |
|  | PE anti-mouse CD4 | Biolegend | 130310 |
|  | PE anti-mouse/human CD45R/B220 | Biolegend | 103208 |
|  | PE anti-mouse/human CD11b | Biolegend | 101208 |
|  | PE anti-mouse Ly-6G/Ly-6C (Gr-1) | Biolegend | 108408 |
|  | PE anti-mouse TER-119/Erythroid Cells | Biolegend | 116208 |
| **Tumor Panel** | BV605 anti-mouse/human CD45R/B220 | Biolegend | 103244 |
|  | PE/Cy7 anti-mouse CD3ε | Biolegend | 100320 |
|  | PE anti-mouse/human CD11b | Biolegend | 101208 |
|  | APC anti-mouse Ly-6G/Ly-6C (Gr-1) | Biolegend | 108412 |
|  | PerCP-cy5.5 anti-mouse CD71 | Biolegend | 113816 |
|  | AF700 anti-mouse CD45.1 | Biolegend | 110724 |
|  | BV650 anti-mouse CD45.2 | Biolegend | 109836 |
|  | APC-ef780 c-Kit (2B8) | eBioscience | 47-1171-82 |
|  | AF647 anti-alpha Tubulin | ABCAM | ab190573 |
|  | AF568 anti-Fibrillarin | ABCAM | ab202540 |
|  | Ki67 APC | Thermo Fisher | 17-5698-82 |

**
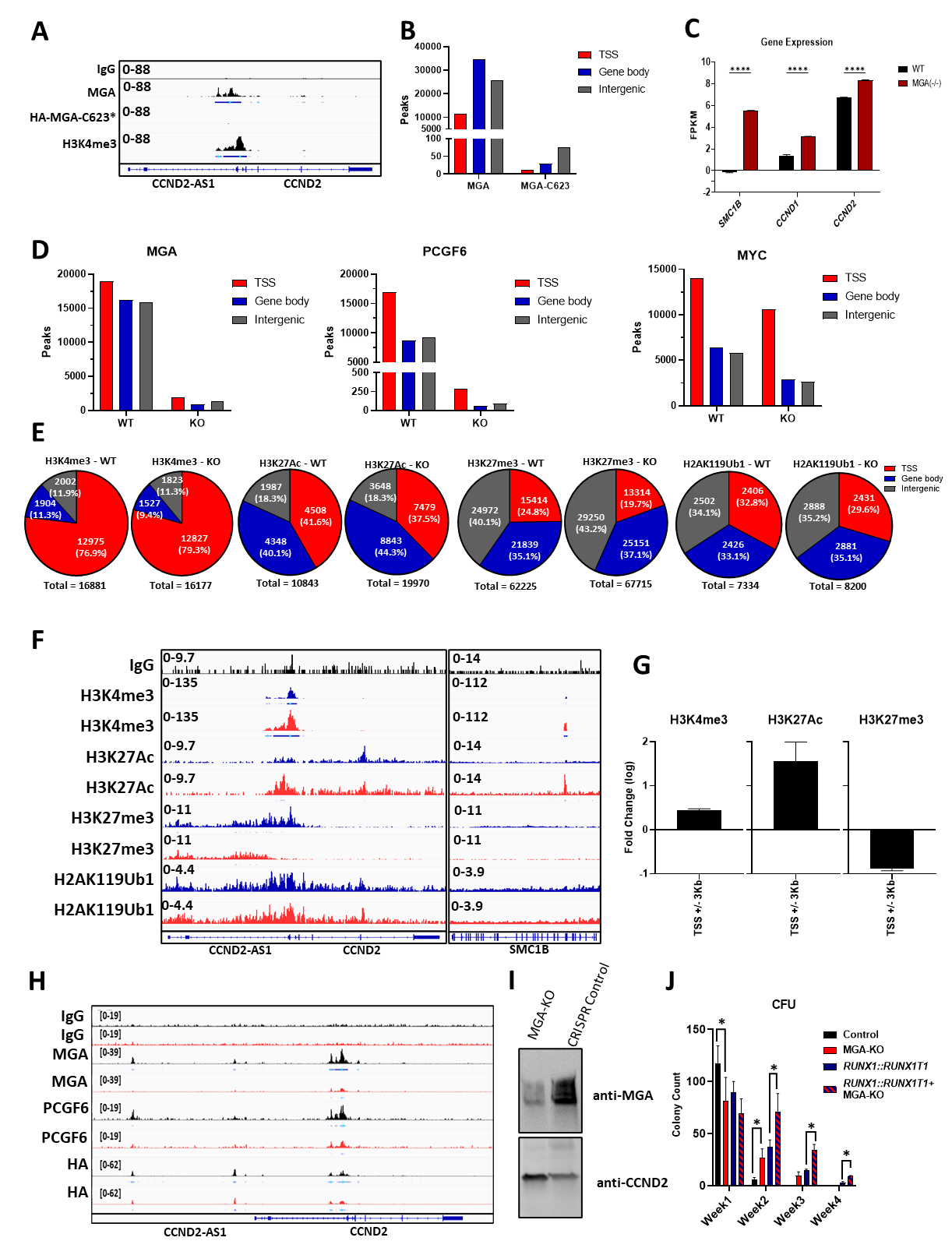
**

**Supplemental Figure 1:**

**A.** CUT&RUN genomic occupancy plots for MGA, HA-MGA-C623*, and H3K4me3 at the *CCND2* locus in MOLM-13 cells expressing HA-MGA-C623*. **B**. Localization of genomic binding peaks for MGA and MGA-C623* in MOLM-13 cells. **C**. Expression of selected upregulated genes in MGA-KO MOLM-13 cells compared to WT controls. Statistics: One-way ANOVA with Bonferroni correction (* p<0.05, ** p<0.01). Error bars indicate the standard error of the mean from three or more biological replicates. **D**. Localization of CUT&RUN peaks for indicated targets in WT and MGA-KO MOLM-13 cells. **E**. Pie chart of the localization of CUT&RUN peaks for different epigenetic marks in WT and MGA-KO MOLM-13 cells. **F**. Coverage plots of various epigenetic marks at the *CCND2* and *SMC1B* loci in WT (blue) and MGA-KO (red) MOLM-13 cells. **G**. MOLM-13 WT vs MGA-KO differential peak analysis for H3K4me3, H3K27Ac, and H3K27me3. **H**. Coverage plots for MGA, PCGF6, and HA-tagged RUNX1::RUNX1T1 genomic occupancy at *CCND2* locus in human CD34 cells transformed by *RUNX1::RUN1T1* that were transduced with an all-in-one MGA-KO CRISPR vector (red) or non-targeting control vector (black) (Addgene #57818). **I**. Western blot analysis of human CD34 cell lysates expressing *RUNX1::RUNX1T1* that were transduced with an all-in-one MGA-KO CRISPR vector or non-targeting control vector. **J.** CFU assay showing the total number of colonies from human CD34 cells expressing *RUNX1::RUNX1T1* from **I**. The all-in-one CRISPR vector backbone was a gift from Benjamin Ebert (Addgene plasmid # 57818). Statistics: (**C**) Two-way ANOVA with Šídák's multiple comparisons test. (**D**) One-way ANOVA with Bonferroni correction (* p<0.05, ** p<0.01). Error bars indicate the standard error of the mean from three or more biological replicates.

**
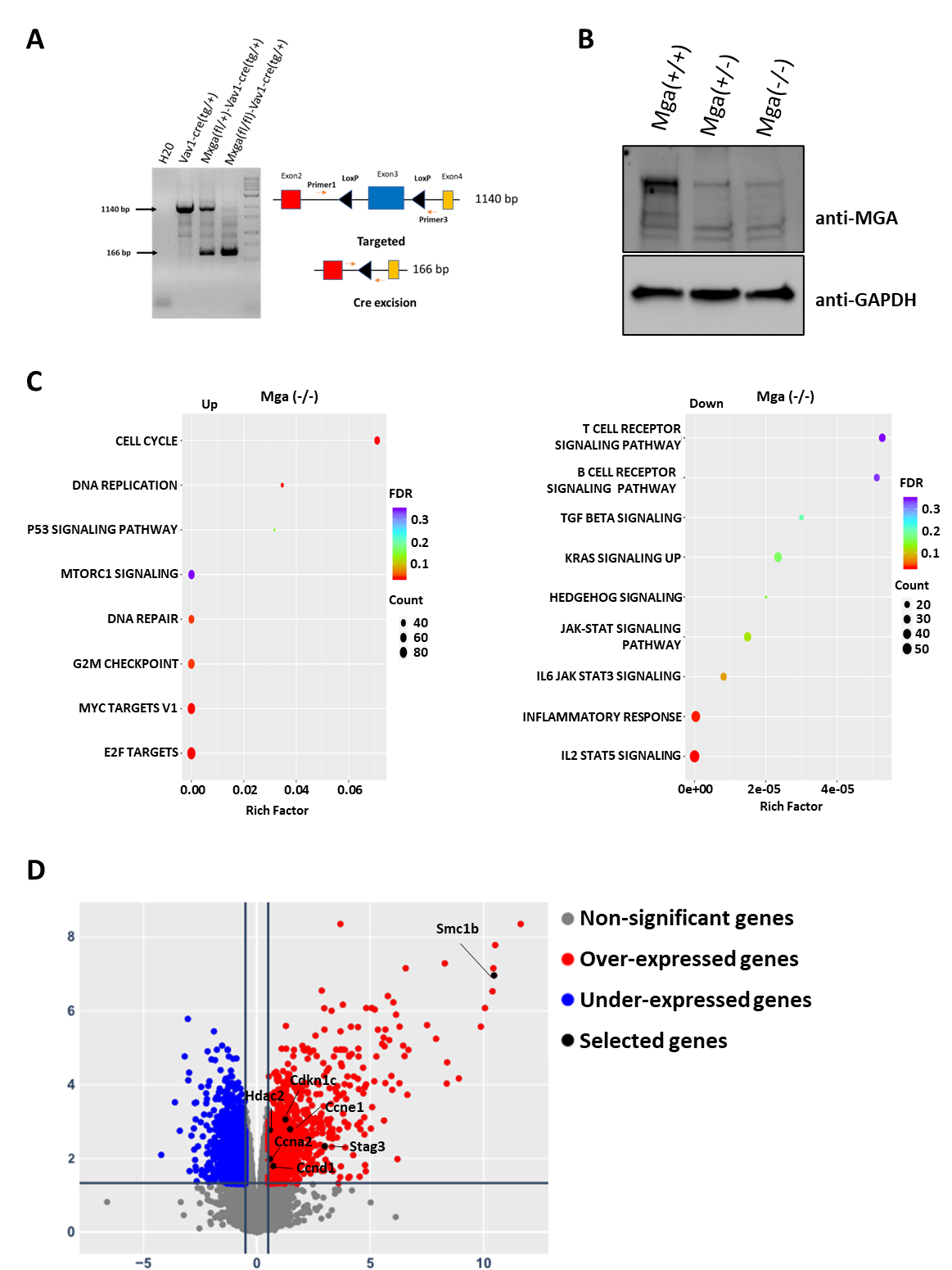
**

**Supplemental Figure 2:**

**A.** Genotyping (left) and schematic (right) showing the conditional knockout mouse model. Exon 3 of *Mga* is flanked by LoxP sites and is removed after Cre expression **B**. Western blot analysis of Mga from Mga(+/+), Mga(+/-), and Mga(-/-) HSPCs isolated from BM. **C**. Rich factor plots of differential gene expression showing up- or downregulated pathways in Mga(-/-) HSPCs. The size of each dot represents gene count, and the color represents FDR. **C**. Volcano plot of differentially expressed genes (DEGs) in Mga(-/-) HSPCs vs. Mga(+/+) HSPCs isolated from BM. Statistics: (**D**) One-way ANOVA with Bonferroni correction (* p<0.05, ** p<0.01). Error bars indicate the standard error of the mean from three or more biological replicates.


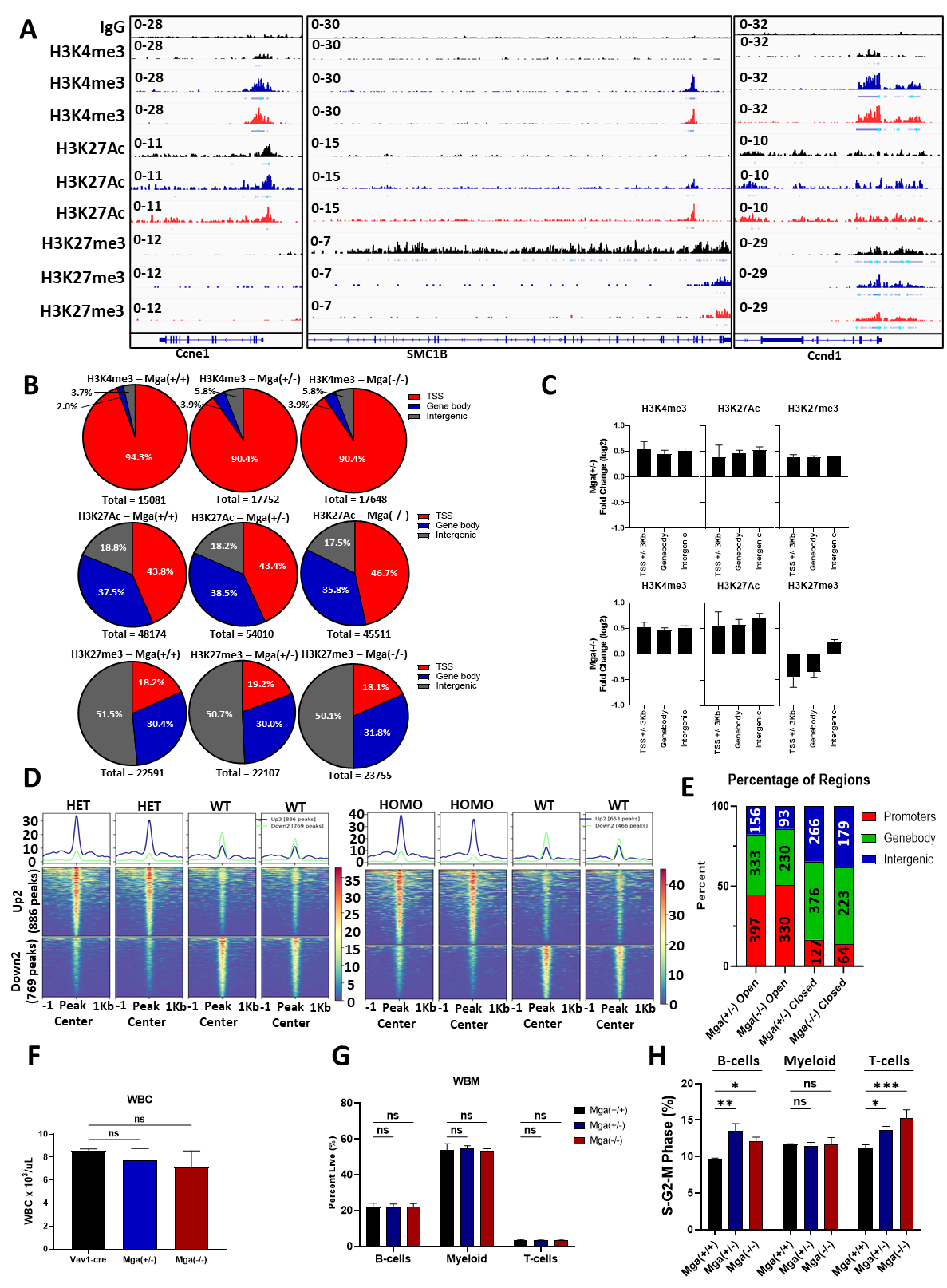


**Supplemental Figure 3:**

**A.** Coverage plots of indicated epigenetic marks on *Ccne1*, *Smc1b*, and *Ccnd1* from WT (black), Mga(+/-) (blue), and Mga(-/-) (red) HSPCs. **B**. Pie chart of localization for indicated epigenetic marks via CUT&RUN from Mga(+/+), Mga(+/-), and Mga(-/-) HSPCs. **C**. Mga(+/+) vs Mga(+/-) and Mga(+/+) vs Mga(-/-) CUT&RUN differential peak analysis for the genomic occupancy of H3K4me3, H3K27Ac, and H3K27me3. Fold change values were selected from genes with a p-value >0.01. **D**. Replicate tornado plots of ATAC-Seq showing the signal enrichment at the most stringent threshold for Mga(+/-) vs. WT (left) and Mga(-/-) vs. WT (right); Up2 and Down2 (FC > 2, FDR < 0.05). **E**. Graph of the genomic context in which the ATAC-seq differential peaks are located using the most stringent threshold Up2 and Down2 (FC > 2, FDR < 0.05) in comparison to WT cells. The values within each section represent the number of regions for each location. Promoter = +/- 2kbp of the TSS; Genebody = Exon, Intron, and TES; Intergenic = all remaining regions. **F**. WBC from WT (n=3), Mga(+/-) (n=3), and Mga(-/-) (n=3) mice at 3 months old. **G**. Flow cytometric analysis of WBM harvested from WT (n=5), Mga(+/-) (n=6), and Mga(-/-) (n=3) mice to characterize mature cell fractions. **H**. Flow cytometric analysis of Ki67/DAPI stained WBM harvested from WT (n=3), Mga(+/-) (n=3), and Mga(-/-) (n=3) mice and stained with conjugated mature antibodies in Supplemental Table 1. F. Statistics: Two-way ANOVA with Dunnett's multiple comparisons test. Error bars indicate the standard error of the mean from three or more biological replicates.


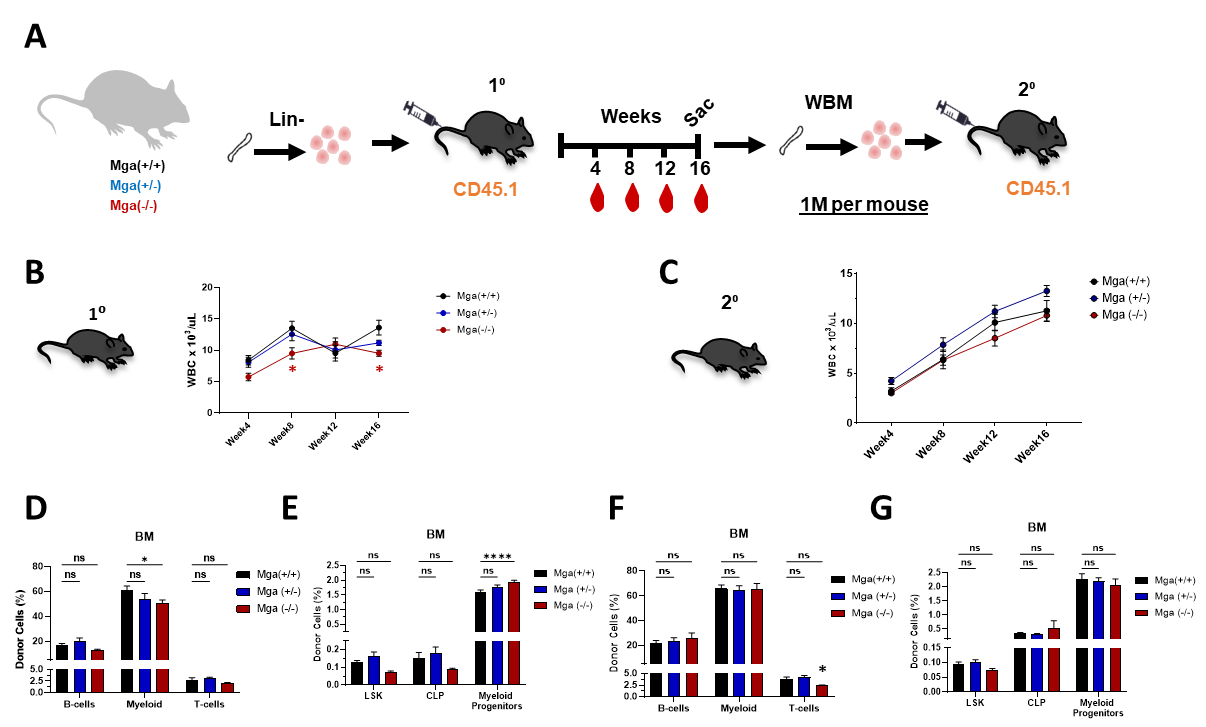


**Supplemental Figure 4:**

**A.** Schematic of the non-competitive serial transplantation model for HSPCs harvested from WT, Mga(+/-), and Mga(-/-) mice. **B-C**. WBC over time from primary (**B**) and secondary (**C**) recipient mice. **D-E**. Flow cytometric analysis of WBM harvested from WT (n=9), Mga(+/-) (n=9), and Mga(-/-) (n=6) primary recipient mice evaluating mature cells (**D**) or HSPCs (**E**). **F-G**. Flow cytometric analysis of WBM harvested from WT (n=9), Mga(+/-) (n=9), and Mga(-/-) (n=6) evaluating mature cells (**F**) or HSPCs (**G**) in secondary recipient mice. Statistics: (**B&C**) One-way ANOVA with Bonferroni correction (* p<0.05, ** p<0.01). Two-way ANOVA with Dunnett's multiple comparisons test. (**D-G**) Error bars indicate the standard error of the mean from three or more biological replicates.

**
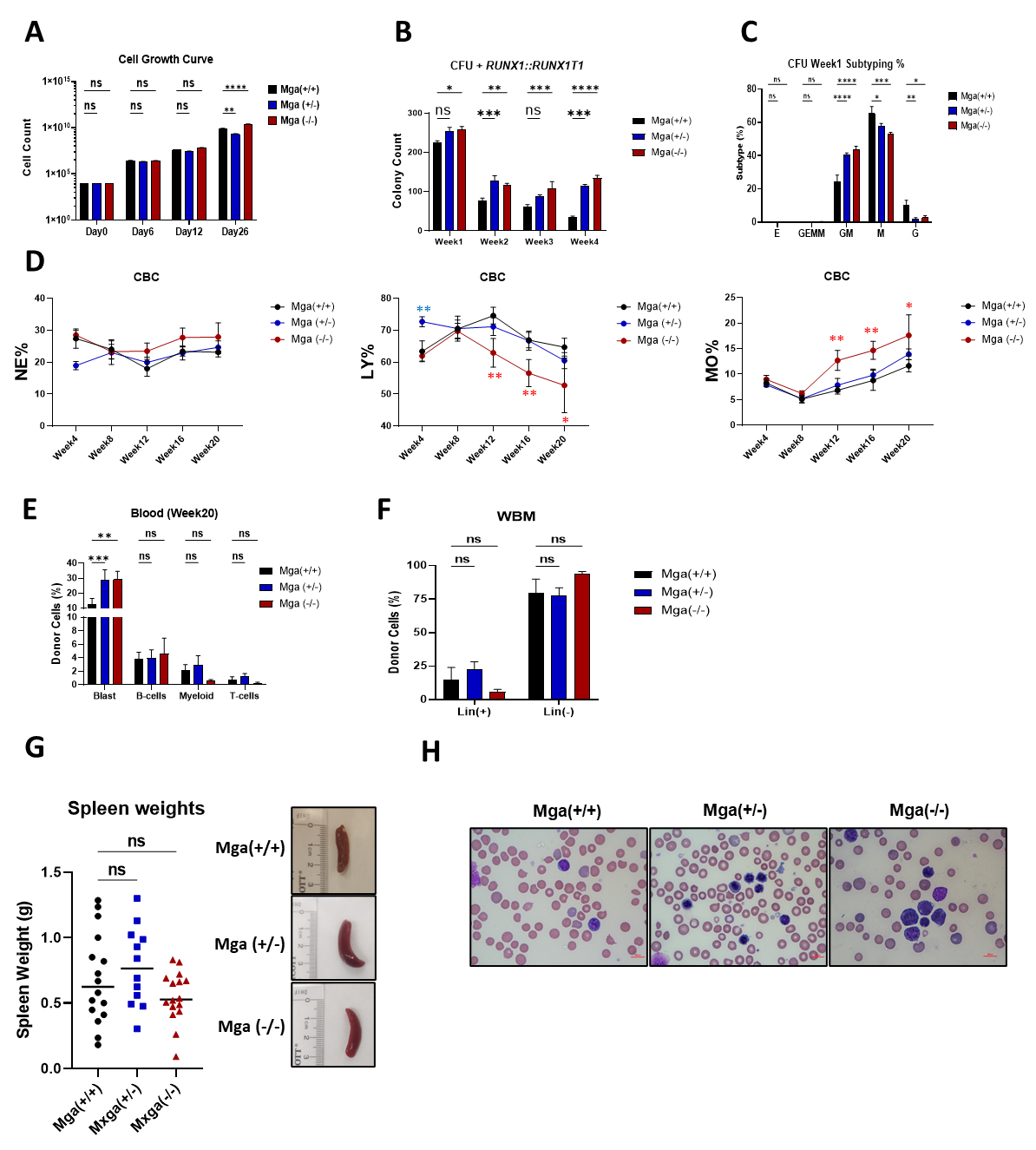
**

**Supplemental Figure 5:**

1. Cell growth of lineage-negative HSPCs transduced with *RUNX1::RUNX1T1* 9A harvested from WT, Mga(+/-), and Mga(-/-) mice maintained in RPMI1640 supplemented with 15% FBS, pen/strep, and murine cytokines including interleukin-3, interleukin-6, SCF, thrombopoietin, and FLT3-L (PeproTech, NJ). **B**. CFU assay showing the total number of colonies from lineage-negative HSPCs from WT (n=9), Mga(+/-) (n=9), and Mga(-/-) (n=9) mice transduced with *RUNX1::RUNX1T1* 9A. **C**. Relative distribution of colony subtypes in (**B**), E = erythroid; GEMM = granulocyte, erythrocyte, monocyte, megakaryocyte; GM = granulocyte, monocyte; M = monocyte; G = granulocyte. **D**. Peripheral blood neutrophils (NE), lymphocytes (LY), and monocytes (MO) percentage over time from recipient mice . **E**. Flow cytometric analysis of peripheral blood harvested from recipient mice at week 20 transplanted with WT (n=6), Mga(+/-) (n=8), and Mga(-/-) (n=4) donor cells transduced *RUNX1::RUNX1T1 9A*. **F**. Flow cytometric analysis of WBM harvested from recipient mice transplanted with WT (n=4), Mga(+/-) (n=5), and Mga(-/-) (n=6) donor cells transduced *RUNX1::RUNX1T1* *9A* measuring maturation; Lin+ = CD11b+, Gr-1+, CD3e+, CD4+. B220+, and Ter119+; Lin- = CD11b-, Gr-1-, CD3e-, CD4-. B220-, and Ter119-. **G**. Spleen weights and size (representative spleens) from recipient mice transplanted with WT (n=16), Mga(+/-)(n=12), and Mga(-/-) (n=16) donor cells transduced *RUNX1::RUNX1T1* 9A. **H**. Representative Wright–Giemsa-stained blood smears at sacrifice. Statistics: (**A-C, E-G**) Two-way ANOVA with Dunnett's multiple comparisons test. (**D**) One-way ANOVA with Bonferroni correction (* p<0.05, ** p<0.01). Error bars indicate the standard error of the mean from three or more biological replicates.


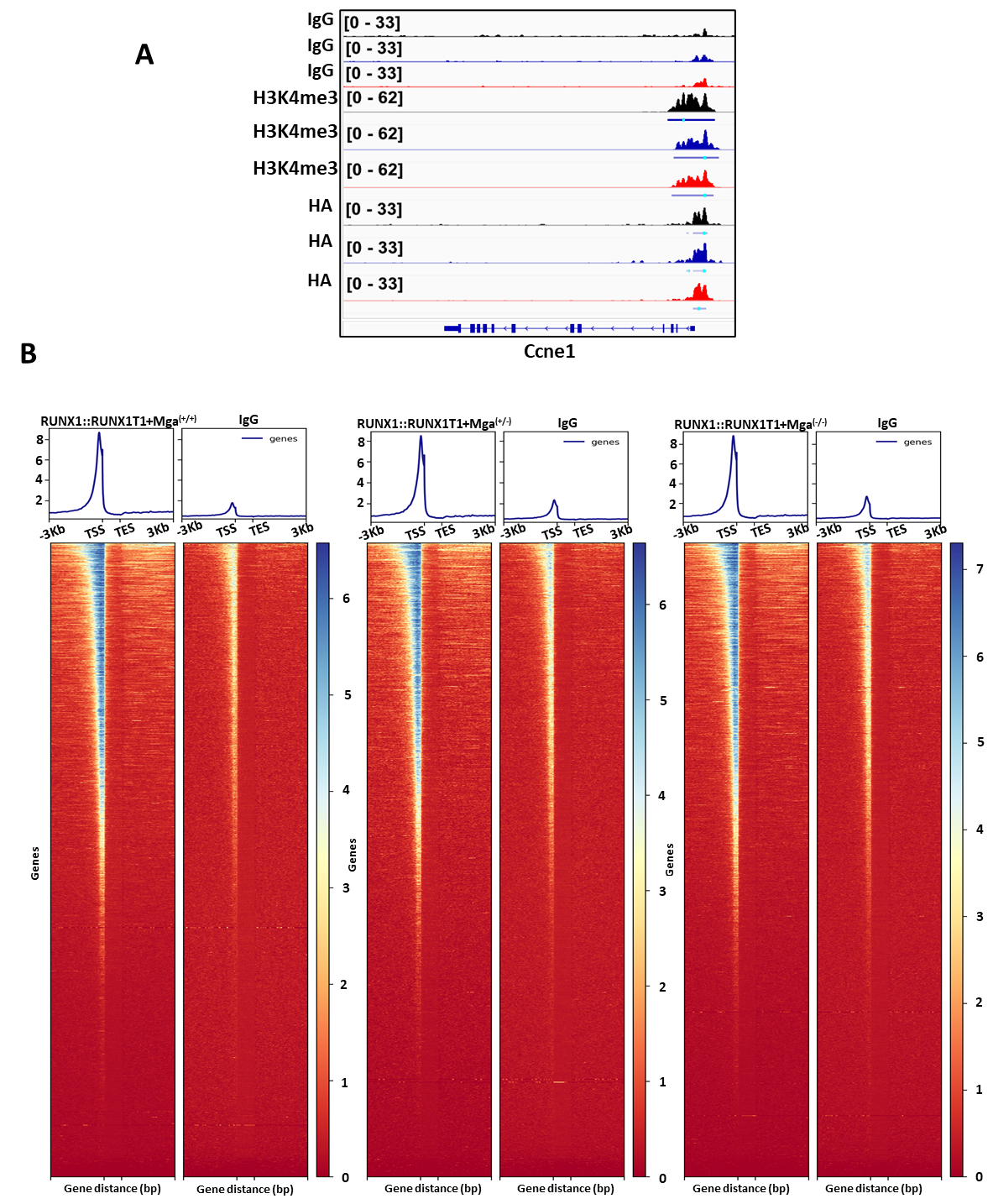


**Supplemental Figure 6:**

**A**. Coverage plots from CUT&RUN of indicated epigenetic marks and HA-tagged RUNX1::RUNX1T1 on *Ccne1* from WT (black), Mga(+/-) (blue), and Mga(-/-) (red) tumors isolated from spleens. **B**. Tornado plot of global RUNX1::RUNX1T1 genomic occupancy.


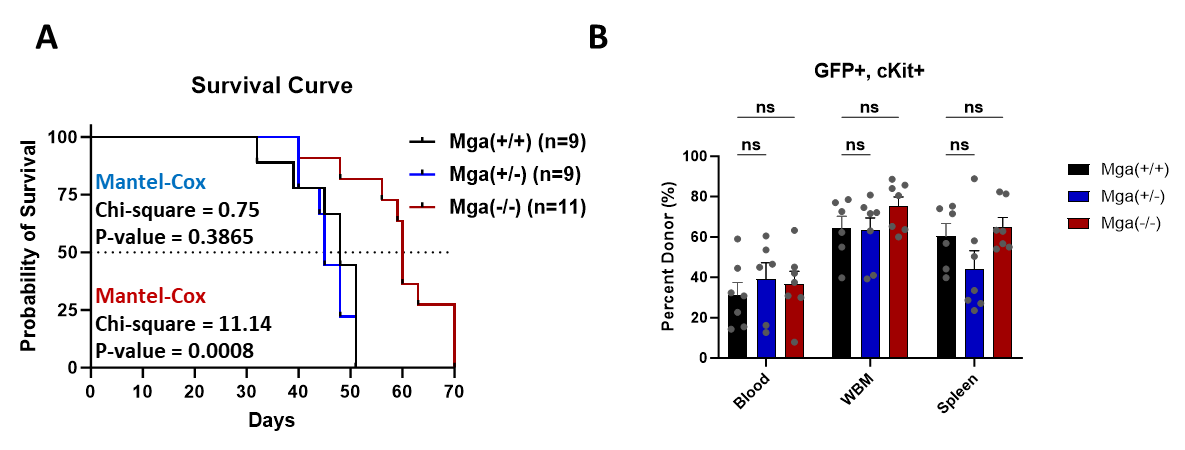


**Supplemental Figure 7:**

**A**. Survival curve of WT, Mga(+/-), and Mga(-/-) *RUNX1::RUNX1T1* 9A secondary recipient mice transplanted with tumors harvested from **Supp. 5H**. Statistics were done using Mantel-Cox test in GraphPad Prism. **B**. Flow cytometric analysis of peripheral blood at week 4 post-transplant and WBM and spleen cells harvested at sacrifice from WT, Mga(+/-), and Mga(-/-) *RUNX1::RUNX1T1 9A* secondary recipient mice showing the expression of GFP+ and c-Kit+ cells (blasts). Statistics: (**B**) Two-way ANOVA with Dunnett's multiple comparisons test. Error bars indicate the standard error of the mean from six or more biological replicates.

**Supplemental References**

1 Thomas, M. E., 3rd *et al.* Pediatric MDS and bone marrow failure-associated germline mutations in SAMD9 and SAMD9L impair multiple pathways in primary hematopoietic cells. *Leukemia* **35**, 3232-3244, doi:10.1038/s41375-021-01212-6 (2021).

2 Schwartz, J. R. *et al.* The genomic landscape of pediatric myelodysplastic syndromes. *Nat Commun* **8**, 1557, doi:10.1038/s41467-017-01590-5 (2017).

3 Heckl, D. *et al.* Generation of mouse models of myeloid malignancy with combinatorial genetic lesions using CRISPR-Cas9 genome editing. *Nat Biotechnol* **32**, 941-946, doi:10.1038/nbt.2951 (2014).

4 Giladi, A. *et al.* Single-cell characterization of haematopoietic progenitors and their trajectories in homeostasis and perturbed haematopoiesis. *Nat Cell Biol* **20**, 836-846, doi:10.1038/s41556-018-0121-4 (2018).

5 Abdelhamed, S. *et al.* Mutant Samd9l expression impairs hematopoiesis and induces bone marrow failure in mice. *J Clin Invest* **132**, doi:10.1172/JCI158869 (2022).
